# Hemiballismus Secondary to Metastatic Lung Cancer: A Case Report

**DOI:** 10.7759/cureus.21454

**Published:** 2022-01-20

**Authors:** Rim Tazi, Zakaria Salimi, Hajar Fadili, Jehanne Aasfara, Asmaa Hazim

**Affiliations:** 1 Neurology, Cheikh Khalifa Ibn Zayed Hospital, Faculty of Medicine, Mohamed VI University of Health Sciences (UM6SS), Casablanca, MAR

**Keywords:** tetrabenazine, the subthalamic nuleus, lung cancer, brain metastases, hemiballismus

## Abstract

Hemiballismus is an unusual complication of brain metastasis of lung cancer. A 62-year-old woman suddenly presented with an acute movement disorder characterized by irregular, involuntary, and large-amplitude movements of the left half of her body. Brain magnetic resonance imaging (MRI) revealed metastasis in the right thalamic region. A whole-body CT scan revealed a lung tumor, while a biopsy showed small cell lung carcinoma. Vascular lesions that affect the basal ganglia, particularly the subthalamic nucleus, are the most common cause of hemiballismus. Hemiballismus is generally treated with antipsychotics such as tetrabenazine and haloperidol, but the primary treatment is the causal one. This was demonstrated in our patient since, after completion of the radio-chemotherapy sessions, the hemiballismus gradually decreased.

## Introduction

The most common sites of brain metastasis from lung cancer are the cerebellum and the frontal and parietal lobes. The main symptoms reported from these locations are ataxia, vertigo, headache, hemiparesis, vomiting, and nausea [1].

Hemiballismus is characterized by irregular, involuntary, and large-amplitude movement of the limbs of half the body controlled by the basal ganglia, especially the subthalamic nucleus. We report hemiballismus as a rare manifestation of lung cancer-derived brain metastasis in the thalamic region.

## Case presentation

We report the case of a 62-year-old woman with no significant medical history. She had been presenting with abnormal movements for several days, especially in her left upper extremity. These movements were accentuated by physical effort and emotions. Moreover, the patient experienced walking difficulties and involuntary movements of the left lower limb. Her doctor prescribed haloperidol, after which the patient was referred to our department.

**Figure 1:**
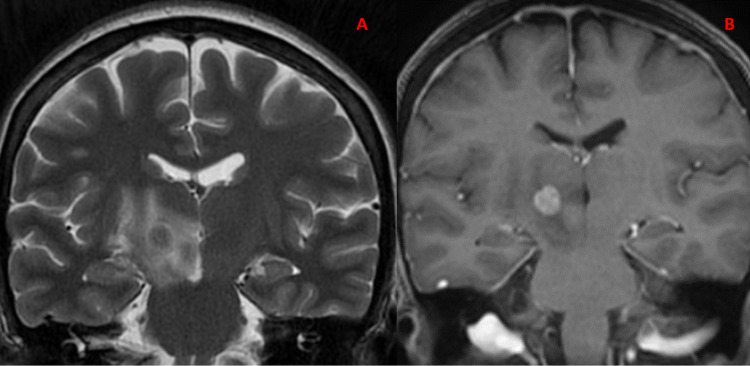
The brain magnetic resonance imaging with contrast shows a lesion of the right thalamic region surrounded by an edema. There is a annular enhancement after injection of the contrast product. (A) T2 coronal section of the brain MRI and (B) enhanced coronal section of the brain MRI.

At admission, the physical examination revealed abnormal movements that were characterized by hyperkinetic, irregular, involuntary, and large amplitude, affecting the left half of her body. Involuntary movements of the face, tongue, and neck were not observed. Brain magnetic resonance imaging (MRI) showed a right thalamic lesion with edema that extended to the subthalamic nucleus. Annular enhancement was observed after injection of the contrast agent (Figure 1).

**Figure 2:**
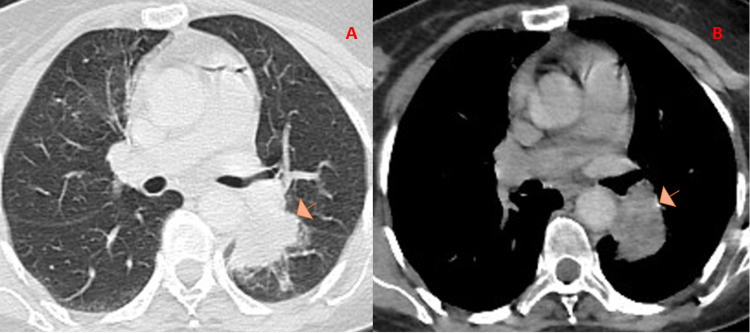
CT chest scan with IV contrast shows a suspected lesion of the left lobe. There is a heterogeneous enhancement after injection of the contrast product. It measured 50 mm on the long axis and 33 mm on the short axis. (A) Lung parenchyma window and (B) bone window CT chest.

Whole-body computed tomography revealed a suspicious lung lesion in the left lobe. This was a proximal lesion with spiculate contours situated in the left lobe. A heterogeneous enhancement was observed after injection of the contrast agent. The lesion measured 50 mm on the long axis and 33 mm on the short axis (Figure 2).

Bronchoscopy with biopsy showed a small cell lung carcinoma. The patient received high doses of haloperidol and corticosteroids but demonstrated no clinical improvement, and thus, the addition of tetrabenazine was then recommended.

After a multidisciplinary discussion, the final diagnosis was T2N0M1 oligometastatic lung carcinoma. The therapeutic management consisted of three sessions of stereotactic and corticosteroid therapy at a rate of 1 mg/kg, after which chemotherapy sessions based on carboplatin and etoposide were administered. The hemiballismus decreased after the radiotherapy sessions, and now she is receiving chemotherapy and has reported clinical improvement.

## Discussion

Hemiballismus is an involuntary movement disorder defined by irregular, violent, and high amplitude movements that involve the ipsilateral arm and leg of one side of the body [2]. Hemiballismus disorder is a consequence of damage to the basal ganglia structures, especially the subthalamic nucleus on the contralateral side of the involved limbs [3]. The Luys body is situated at the junction of the diencephalon and midbrain next to the thalamus and the red nucleus [4]. Hemiballismus is the consequence of a lesion in the contralateral Luys body, but it may also result from damage to its projections or even to the structures with which it shares connections, especially the striatum, globus pallidus, thalamus, primary motor cortex, and primary somatosensory cortex [5]. In the physiological state, the neurons of the nucleus of Luys will stimulate the globus pallidus and support thalamic inhibition. Therefore, hemiballismus is the consequence of disinhibited thalamic activation when the activity of the subthalamus is interrupted [6]. The most common causes of hemiballismus are ischemic or hemorrhagic strokes, while brain metastasis is very rare [7]. Here, we report a case of hemiballismus secondary to lung cancer-derived brain metastasis.

Treatment of the underlying etiology is very important and leads to a decrease in hemiballismus over time. Hemiballismus is treated with antipsychotics, such as haloperidol, which we administered to our patient with no clinical improvement; thus, the addition of tetrabenazine was recommended. Tetrabenazine is the standard treatment for abnormal movements, particularly hemichorea and hemiballismus [8]. This agent acts at the level of the dopaminergic synapse by inhibiting the central vesicular transporters (VMAT2). This promotes a decrease in the dopamine concentration in the central nervous system, which increases the difficulty of performing movements controlled by the primary motor cortex [8].

Corticosteroid therapy controls symptoms by reducing perilesional tumor edema [9]. The efficacy of this therapy was demonstrated in the case reported by Fraser, but our patient did not report any improvement after corticosteroid treatment. Rather, a decrease in her abnormal movements was observed after radio-chemotherapy. This could be explained by the resolution of the edema around the right thalamus and the reperfusion of the damaged tissue.

The article discusses a rare case presentation of hemiballismus due to brain metastatic lung cancer. Hemiballismus was secondary to the thalamic metastatic site rather than the paraneoplastic effect of lung cancer. As reported in the literature, brain metastases are very common with small cell lung cancer as well as non-small cell lung cancer [1]. Our case fits within the existing literature. Its illustrations show brain metastases from small-cell lung cancer. It also highlights the importance of early diagnosis and the efficacy of radio-chemotherapy to reduce hemiballismus over time.

## Conclusions

Hemiballismus may be the first clinical manifestation of brain metastases of lung cancer. Brain MRI should be performed at baseline to confirm the presence of brain metastasis in the contralateral thalamic or subthalamic nucleus. We stress that causal treatment based on radio-chemotherapy decreases abnormal movements. Our case indicates that an early diagnosis of lung cancer and effective treatment may improve the quality of life of patients.
